# Detergent-resistant α-amylase derived from *Anoxybacillus karvacharensis* K1 and its production based on whey

**DOI:** 10.1038/s41598-024-63606-7

**Published:** 2024-06-03

**Authors:** Diana Ghevondyan, Tigran Soghomonyan, Pargev Hovhannisyan, Armine Margaryan, Ani Paloyan, Nils-Kåre Birkeland, Garabed Antranikian, Hovik Panosyan

**Affiliations:** 1 Department of Biochemistry, Microbiology and Biotechnology, Yerevan State University, Alex Manoogian 1, 0025 Yerevan, Armenia; 2 Biology Faculty, Research Institute of Biology, Yerevan State University, Alex Manoogian 1, 0025 Yerevan, Armenia; 3 Laboratory of Protein Technologies, Scientific and Production Center “Armbiotechnology” NAS RA, 0056 Yerevan, Armenia; 4 Department of Biological Sciences, University of Bergen, NO-5020 Bergen, Norway; 5 Center of Biobased Solutions (CBBS), Institute of Technical Biocatalysis, Hamburg University of Technology, 21073 Hamburg, Germany; 6 Present Address: Department of Microbiology, Biocenter, University of Wuerzburg, 97074 Wuerzburg, Germany

**Keywords:** α-amylase, *Anoxybacillus karvacharensis*, Cloning, Expression, Biochemical characterization, Acid whey, Biochemistry, Biotechnology, Microbiology, Molecular biology

## Abstract

In the field of biotechnology, the utilization of agro-industrial waste for generating high-value products, such as microbial biomass and enzymes, holds significant importance. This study aimed to produce recombinant α-amylase from *Anoxybacillus karvacharensis* strain K1, utilizing whey as an useful growth medium. The purified hexahistidine-tagged α-amylase exhibited remarkable homogeneity, boasting a specific activity of 1069.2 U mg^−1^. The enzyme displayed its peak activity at 55 °C and pH 6.5, retaining approximately 70% of its activity even after 3 h of incubation at 55 °C. Its molecular weight, as determined via SDS-PAGE, was approximately 69 kDa. The α-amylase demonstrated high activity against wheat starch (1648.8 ± 16.8 U mg^−1^) while exhibiting comparatively lower activity towards cyclodextrins and amylose (≤ 200.2 ± 16.2 U mg^−1^). It exhibited exceptional tolerance to salt, withstanding concentrations of up to 2.5 M. Interestingly, metal ions and detergents such as sodium dodecyl sulfate (SDS), Triton 100, Triton 40, and Tween 80, 5,5ʹ-dithio-bis-[2-nitrobenzoic acid (DNTB), β-mercaptoethanol (ME), and dithiothreitol (DTT) had no significant inhibitory effect on the enzyme’s activity, and the presence of CaCl_2_ (2 mM) even led to a slight activation of the recombinant enzyme (1.4 times). The Michaelis constant (*K*_*m*_) and maximum reaction rate (*V*_*max*_), were determined using soluble starch as a substrate, yielding values of 1.2 ± 0.19 mg mL^−1^ and 1580.3 ± 183.7 μmol mg^−1^ protein min^−1^, respectively. Notably, the most favorable conditions for biomass and recombinant α-amylase production were achieved through the treatment of acid whey with β-glucosidase for 24 h.

## Introduction

α-Amylase (EC3.2.1.1, 1,4-α-D-glucan glucanohydrolase, glycogenase, and endoamylase) is a metalloenzyme able to hydrolyzes starch and other α-glucans by spliting internal α-1,4-glucosidic linkages to glucose, maltose, maltotriose, dextrin, and branched oligosaccharides containing 6–8 glucose units^1,2^. Amylases are among the most relevant enzymes widly used in many industrial processes. Currently, genetically modified α-amylases have many applications in the detergent, bakery, beer, textile, paper, pulp, and pharmaceutical industries^3,4^.

Several microorganisms produce amylolytic enzymes. Amylases derived from plants, fungi, bacteria, and archaea have been used in the brewing industry for the oldest time.

Among the different types of α-amylases, those adapted to heat are superior to their mesophilic counterparts because of their high thermostability and activity under harsh processing conditions. Thermophilic microbes are phylogenetically very diverse and are mainly represented by bacteria and archaea^5,6^. Among the thermophilic bacteria, many *Anoxybacillus* species have been recognized as active producers of thermostable α-amylase satisfying industrial requirements^2,7,8^.

The production of recombinant enzymes is one of the main pillars of modern biotechnology. Recombinant enzymes are mainly used for the production of pure and functional target molecules applicable in subsequent processes. Over the last decade, the overproduction of recombinant thermostable α-amylases has been widely carried out^9,10^).

The concept of a bio-based circular economy has come to the forefront in the past decade, aiming to develop sustainable biotechnological processes and reduce the demand for fossil fuels^11^. In recent years, researchers have been focused on microbes adapted to extreme conditions to find out their biotechnological significance. Because of their unique genomic resources, extremophiles play a significant role globally as technological drivers^11^.

Bioconversion of agro-industrial wastes to produce microbial biomass and recombinant enzymes has important applications in biotechnology via the circular economy approach, offering both economic and environmental benefits^12,13^. Whey as a high nutritional value waste can be used to obtain microbial biomass and value-added products, including chemical compounds, polymers and enzymes^14^.

To make whey a more attractive growth medium, however, additional treatments are required. This allows the development of a more efficient fermentation process, leading to high cell density and high enzyme production during fermentation. Cost-effective production of robust amylases is still greatly needed for various industrial processes. In the present study, an α-amylase gene from the recently isolated alkali-tolerant thermophile *Anoxybacillus karvacharensis* K1 was cloned and expressed in *Escherichia coli* BL21 (DE3) cells. The purification and characterization of recombinant α-amylase and bioconversion of whey for the production of microbial biomass and recombinant α-amylase, were also reported.

## Materials and methods

### Strain culture conditions and α-amylase production

*A. karvacharensis* K1 (DSM 106524^ T^, KCTC 15807^ T^) was recently isolated from a water–sediment slurry sample obtained from a geothermal spring at Karvachar (Nagorno-Karabakh, 40°17′41.00″ N, 46°27′50.00″ E, 1584 m altitude), and fully characterized^8^. A 24-h culture was streaked on nutrient starch plates (g L^−1^: soluble starch, 20.0; peptone, 10.0; KH_2_PO_4_, 5.0; agar, 15.0; pH 7.0) for further determination of amylolytic activity by incubating it at 60 °C. After 24 h of incubation, the plates were treated by Lugol’s solution (w v^−1^(%): I_2_, 0.5_;_ KI, 1.0; distilled water) to determine the clearance zone against the starch plate as the control. The strain was routinely maintained in a solidified nutrient broth (pH 7.0) medium slants. The stock culture was stored in 40% (v v^−1^) glycerol at − 80 °C.

*A. karvacharensis* K1 was grown in a medium containing (g L^−1^): soluble starch, 20.0; yeast extract, 10.0; peptone, 10.0; KH_2_PO_4_, 5.0; NaCl, 5.0, MgSO_4_, 5.0; and CaCl_2_, 5.0; pH 7.0–7.2. The inoculated medium was incubated at 60 °C in an orbital shaker at 160 rpm. The effects of incubation temperature and pH of the medium on enzyme production were studied. To determine the effect of nitrogen sources on enzyme production, yeast extract, beef extract, tryptone, casein hydrolysate, and peptone at concentrations of 5–15 g L^−1^, as well as ammonium sulfate and ammonium nitrate at concentrations of 0.1–1 g L^−1^ were used.

To produce biomass bacteria were inoculated in the medium with the highest α-amylase production and incubated at 60 °C and 160 rpm. Production was continued for 18 h by collecting samples every hour, and bacterial growth was measured by spectrophotometry at OD_600_. After centrifugation of the samples at 10,000 rpm, the enzyme activity in the supernatant was examined by Nelson-Somogyi method^15^.

### Sequence analysis and amplification

The α-amylase sequence was derived from the draft genome of *A. karvacharensis* K1 assigned to GenBank under the accession number WP 143,271,304. Multiple sequence alignment of the α-amylase from *A. karvacharensis* K1 with other α-amylase sequences was performed using the Clustal Omega Multiple Sequence Alignment web tool (https://www.ebi.ac.uk/Tools/msa/clustalo). The evolutionary history was inferred using the Maximum Likelihood method and JTT matrix-based model^16^. Evolutionary analyses were conducted using MEGA X^17^. A 3D structural model of α-amylase was constructed using the SWISS-MODEL web tool (https://swissmodel.expasy.org). The 3D structure was analyzed using the PyMOL software. The HMMER web server (https://www.ebi.ac.uk/Tools/hmmer/) was used to annotate the functional domains in the α-amylase protein sequence based on the Pfam database.

The α-amylase gene sequence (locus_tag BO219_13480) was retrieved from the annotated strain genome. A primer set for gene amplification and cloning was designed using the FX Cloning Primer Design Tool (http://www.fxcloning.org/). Restriction sites for the NdeI and XhoI restriction endonucleases were also included. The GenElute™ Bacterial Genomic DNA Kit (Sigma) was used to isolate genomic DNA. The Thermo Scientific™ Genomic DNA Purification Kit was used to purify isolated genomic DNA. The purified DNA was used as a template for PCR amplification of the α-amylase coding sequence, starting from the putative signal peptide cleavage site down to the stop codon, using the designed oligonucleotides AmyF (5′-CATGGCTAGCATGAATAATGTGAAAAAAGTATGGTTGTATTATTCTATAATTGC) and AmyR (5′-CATGCTCGAGTGGCACATTCCAACTAGCGG) (the restriction sites are highlighted). The amplification mixture (50 μL) contained ~ 100 ng DNA, 10 mμL 5 × Phusion Reaction Buffer (BioLabs, New England), 0.5 μL Phusion DNA polymerase (BioLabs, New England), 1 μL 10 mM dNTPs (Sigma), and 2.5 μL of a 10 μM stock solution of each primer. The following amplification program was used: initial denaturation at 98 °C for 30 s, followed by 30 cycles of denaturation at 98 °C for 10 s, annealing at 68 °C for 20 s, extension at 72 °C for 1 min, and final extension at 72 °C for 5 min, after which the reaction was cooled to 4 °C. The PCR products were analyzed by electrophoresis with 1.0% agarose gel. 1 Kb Plus DNA Ladder was loaded along with the PCR products. The PCR products were purified using the GenElute PCR Cleanup Kit (Sigma).

### Cloning, expression, and recombinant enzyme purification

The gene was cloned into the p7XNH3 expression vector (Novagen, USA) and expressed in *E. coli* BL21 (DE3) cells (Thermo Fisher Scientific).

The purified PCR product and plasmid vector were digested with NdeI and XhoI endonucleases (Ipswich, MA, USA) in the reaction mixture (final volume of 10 μL) containing the insert and the vector in the molar ratio of 1:5, 1 μL of the appropriate restriction enzyme, 1 μL rCutSmart Buffer (NEB), and water at 37 °C for 1 h. After heat inactivation at 65 °C for 20 min, 0.8 μL T4 DNA Ligase (NEB) and 1.2 μL 10 × T4 DNA Ligase Buffer (NEB) were added to the reaction mixture. The mixture was incubated for 1 h at 20 °C. After incubation, 1 μL of the mixture was used to transform the chemically competent One Shot *E. coli* BL21 (DE3) cells (Thermo Fisher Scientific) according to the protocol. The constructed pET-21b vector was transformed into *E. coli* BL21 (DE3) competent cells, and the p7XNH3 plasmid was prepared from a single clone.

Plasmid resistance against kanamycin was used as a selective marker to obtain positive bacterial clones on a Luria–Bertani (LB) agar medium supplemented with kanamycin (100 μg mL^−1^). The p7XNH3 plasmid gene sequence was verified by DNA sequencing using the T7 promoter and T7 terminator primers.

For the gene expression analysis, *E. coli* BL21 (DE3) cells harboring the recombinant p7XNH3 plasmids were first inoculated into the LB medium supplemented with 100 μg mL^−1^ kanamycin and incubated at 37 °C and 180 rpm overnight. After the OD_600_ reached 0.6, 1 mM isopropyl-β-D-thiogalactoside (IPTG) was added to the culture medium to induce recombinant protein production. The mixture was incubated at 18 °C and 180 rpm for 24 h, then the cells were harvested by centrifugation (10,000 × g and 4 °C for 20 min), and the obtained pellets were stored at − 20 °C for further experiments. The cells (1–5 g) were resuspended in 5 mL of the ice-cold lysis buffer (50 mM KH_2_PO_4_ pH 8.0; 300 mM NaCl; 10 mM imidazole). The cell suspension was sonicated at medium intensity for 10 min (30 s pulse, 30 s interval) in a falcon tube while stirring on ice using a sonicator (Labsonic 2000, B. Braun Germany). The sonicated cell lysate was centrifuged at 1000 × g at 4 °C for 40 min, followed by supernatant filtration with 0.2 μm filters. Subsequently, the polyhistidine-tagged recombinant enzyme was purified from the cleared supernatant by affinity chromatography using HisTalon Gravity Columns (Clontech) according to the manufacturer’s protocol. Hexahistidine-tagged α-amylase was purified to homogeneity using nickel affinity chromatography with Profinity IMAC Ni-charged resin (Bio-Rad). The column was equilibrated with a wash buffer (50 mM KH_2_PO_4_, pH 8.0; 300 mM NaCl; 10 mM imidazole). The supernatant was transferred to a column and incubated for 60 min at 4 °C. After removing the flow through from the column, the resin was washed twice with 4 mL of the washing buffer, and the bound protein was eluted four times with 1 mL of the elution buffer (50 mM KH_2_PO_4_ pH 8.0, 300 mM NaCl; 500 mM imidazole). The sodium dodecyl sulfate–polyacrylamide gel electrophoresis (SDS-PAGE) was used to analyze of the all fractions. The eluted fractions containing the targeted protein were buffer exchanged to remove the imidazole, concentrated using 30 kDa cut-off protein concentrators (Merck Amicon™), and stored at − 20 °C in 50% glycerol for further analyses. The protein concentration was measured by the Bradford method using bovine serum albumin as the standard^18^.

### Enzyme activity assay

The α-amylase activity was determined by detecting the amount of liberated reducing sugars, as described by Nelson^15^. In brief, about 200 μL of the reaction mixture containing 1% soluble starch and 0.1 M acetic acid–sodium buffer (pH 6.0) were incubated at 55 °C for 10 min. The reaction was initiated by adding the enzyme solution. After reacting for 10 min, 800 μL of deionized water and 1 mL of Somogyi reagent were added and boiled in a water bath for 10 min. The liberated reducing sugars were estimated by adding 1 mL of Nelson’s reagent, followed by cooling. The reducing sugars were measured by a spectrophotometer at λ 560 nm using glucose as the standard. The amount of enzyme able to release 1 μmol of reducing sugar per minute under the assay conditions was considered as a one unit of α-amylase.

### Molecular mass determination and zymogram preparation

SDS-PAGE was applied as described by Laemmli^19^ using 12% polyacrylamide. Electrophoresis was performed using a PROTEAN II xi cell (Bio-Rad). The molecular weight of the enzyme was estimated by running the Amersham ECL Rainbow Marker (Full range, Merck) and a 10–180 kDa Triple Color Protein Marker (BioFACT) on the samples. The stain Coomassie brilliant blue R 250 was used for visualization of the protein bands.

A zymogram of α-amylase was obtained using 7.5% native PAGE with 0.25% (w v^−1^) soluble starch. The obtained gel was washed with deionized water and incubated in phosphate-buffered saline (pH 7.0) at 55 °C for 30 min. Subsequently, it was stained with an iodine solution for 5 min at room temperature. The appearance of clear yellow zone on the dark blue background of stained starch was used to detect the α-amylase band^20^.

### Characterization of the recombinant enzyme

#### Effect of temperature and pH on enzyme activity

The temperature profile was determined by measuring the enzyme activity under the described conditions at temperatures ranging from 25 to 80 °C with 5 °C intervals. The enzyme thermostability was evaluated by pre-incubating it at different temperatures (50, 55, and 60 °C) for 60–180 min, followed by incubation on ice. The residual activities were determined under the above assay conditions.

The effect of pH on α-amylase activity was determined by measuring the enzyme activity in the pH range of 3–12 with the Britton–Robinson buffer by incubating at 55 °C. In addition, the effect of buffers on the enzyme activity was measured in Britton–Robinson, Phosphate, Mops, and Hepes buffers at pH 6.0, 6.5, and 7.0^21^.

#### Effects of metal ions and denaturing chemicals on enzyme activity

The effects of various metal ions on enzyme activity were measured by pre-incubating the enzyme with 2 mM metal solutions of KCl, CuSO_4_, K_2_SO_4_, CoSO_4_, Na_2_S, NiCl_2_, MnSO_4_, BaCl_2_, FeSO_4_, MgCl_2_, ZnSO_4_, FeCl_3_, MgSO_4_, and CdCl_2_ without a substrate for 120 min at 4 °C. To determine the effect of NaCl concentration on the enzyme activity NaCl at a concentration of 0.5–2.5 M were added to the reaction mixture and incubated. The residual activity was measured under optimum reaction conditions. The effect of Ca^2+^ ions on the enzyme activity was determined by adding CaCl_2_ to the reaction mixture at concentrations ranging from 1 to 10 mM. Enzyme activity was determined under the optimum reaction conditions.

The effects of detergents (1%), such as SDS, Triton 100, Triton 40, and Tween 80, and reducing agents, such as 5,5ʹ-dithio-bis-[2-nitrobenzoic acid (DNTB, 5 mM), β-mercaptoethanol (ME, 5 mM), and dithiothreitol (DTT, 2 mM), on the enzyme activity were also determined. The purified enzyme was incubated with the aforementioned compounds for 120 min at 4 °C. In the study of ethylenediaminetetraacetic acid (EDTA), the enzyme was incubated with 10 mM EDTA overnight at 4 °C.

The control was prepared in the absence of the test compounds. All experiments were performed twice.

#### Substrate specificity

The potato, rice, wheat, and corn starches, as well as amylose, amylopectin, and α-, β-, and γ- cyclodextrins (1% concentration), in their native forms were used to find out the substrate specificity of the enzyme. The hydrolysis was carried out at optimum temperature and pH conditions and 1% (w v^−1^) substrate concentration. The reducing sugars released after hydrolysis were determined by Nelson-Somogyi method^15^.

#### Kinetic parameters

The kinetic parameters were determined by performing a standard assay with 12 different soluble starch concentrations varying from 0 to 10 g L^−1^. The Michaelis constant (*K*_m_) and maximum reaction rate (*V*_max_) were calculated by nonlinear regression using the Michaelis–Menten equation^22,23^. Subsequently, the values of the corresponding kinetic parameters and their standard errors were calculated by multivariate linear regression analysis using software developed on the Gauss 4.0 language^24^.

#### Enzyme stability against protease

To check enzyme stability against to proteases 10 U Proteinase K (SIGMA, USA) was added on the partially purified amylase and mixture was incubated at room temperature for 30 min. The remaining amylase activity was determined by assaying the reducing sugars using Nelson-Somogyi method^15^.

#### Analysis of the hydrolysis products

The end products after hydrolysis of soluble starch derived from potato, rice, and wheat (1%) were determined by incubating the starch with α-amylase in standard reaction mixtures at 55 °C and pH 6.5. After incubation for 3 h, the reaction mixture was placed in ice bath aiming to stope the enzymatic reaction. The hydrolysis products were detected by high-performance liquid chromatography (HPLC) furnitured by a high-performance carbohydrate column (Su-Spher Si-100 NH2, 250 mm × 4.6 mm) at 30 °C and 4.45 MPa pressure, with acetonitrile and water mixture (75:25) as the mobile carrier at a flow rate of 1.4 mL min^−1^, and detected by a Waters 2414 refractive index detector. HPLC-grade glucose and maltose were used as the standards.

### Utilization of dairy industry waste as a substrate for recombinant *E. coli*

To estimate the growth rate and α-amylase production by recombinant *E. coli* BL21 (DE3), the strain was inoculated separately into two different liquid basal media based on acid (AW) and sweet whey (SW). To prepare the media, AW and SW were diluted two times and adjusted to a pH of 7.5 with NaOH and H_2_SO_4_. These media were used in their intact and enzymatically treated forms (designated as AW_E_ and SW_E_, respectively). Enzymatic treatment was carried out using β-glucosidase cloned and characterized from a hot spring metagenome^25^. For the enzymatic treatment, AW and SW were diluted twice (1 L each) and incubated at 80 °C for 30 min. Aliquots of reaction mixture were taken at 10 min intervals to estimate lactose hydrolysis.

Lactose concentrations in AW and SW were measured using the Nelson–Somogyi method^15^ considering that lactose is the only sugar present in whey. The glucose concentration after enzymatic treatment was estimated using the coupled glucose oxidase–peroxidase assay procedure described by Haider and Husain^26^, with a slight modification. In this experiment we used solution A containing 0.3 mM 4-amino-antipyrine, 5 mM phenol, 100 mM Na-acetate pH 5.8, 1 U peroxidase, and 1 U glucose oxidase. The glucose oxidase was purified from *Penicillium chrysogenum,* while peroxidase was purified from horseradish. 1.5 mL of the solution A was added to all assay tubes. To measure the β-D-glucose concentration after lactose hydrolysis the defined amounts of SW and AW pretreated β-glucosidase were used. The tubes were incubated at 30 °C for 10 min, and then the measurement was carried out spectrophotometrically by adsorption at OD_550_. The 5 mM β-D-glucose was used as a standard. The calculation was done based on the following Eq. (1):

1 $$C = \frac{{A_{Sample} }}{{A_{standard} }} \times C_{standard}$$

where A_sample_ is the adsorption of the sample, A_standard_ is the adsorption of the β-D-glucose standard, and C_standard_ is the concentration of β-D-glucose (mM).

The media were then treated at 105 °C for 20 min, filtered through cotton filters and filter paper, and boiled for 10 min. Kanamycin was added to the media at a concentration of 100 μg mL^−1^. Nutrient Broth medium was used as the control medium. A 2% inoculum was added to each medium and incubated at 37 °C for 24 h at 150 rpm (IKA KS 4200i control shaker). Samples were collected after 9, 17, and 24 h of growth. Bacterial growth was spectrophotometrically estimated at OD_600_. The cells harvested by centrifugation (10,000 × g at 4 °C for 20 min), were sonicated, and their α-amylase activity was measured.

### Statistical analysis

The obtained data in this study were from at least two independent experiments, each of them with two biological repeats. The data were expressed as means ± SE (standard errors) unless otherwise denoted.

## Results

### Enzyme production

*A. karvacharensis* K1 was found to be a potent amylase producer, showing a clearance zone on the starch plate after treatment by Lugol’s solution (Fig. S1).

The growth of the strain and the production of the α-amylase were observed at the temperature and pH ranges of 45–80 °C and 5.5–8.5, respectively. The highest production of α-amylase was observed at its optimum growth temperature of 60 °C and pH of 7.0. The enzyme production was associated with growth. It began in the early log phase and reached its maximum value in the late exponential phase (Fig. S2).

The maximum enzyme production was found in the medium containing 1% (w v^−1^) yeast extract and peptone as sources of nitrogen. Among the inorganic nitrogen sources (0.5% w v^−1^), only ammonium nitrate enhanced amylase production.

### Gene sequence analysis and amplification

**Figure 1:**
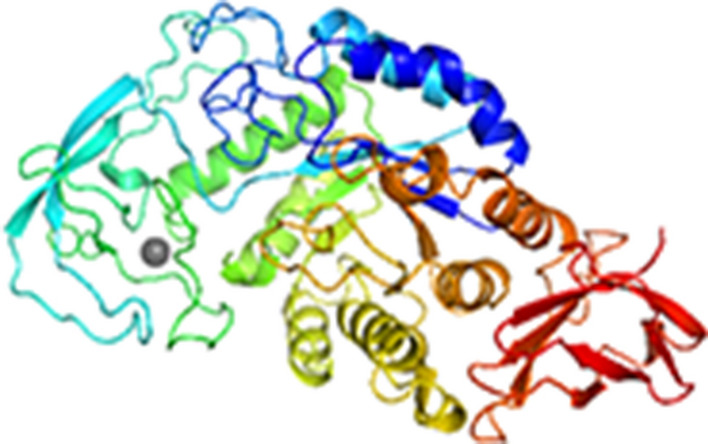
The 3D structure modeling of *A. karvacharensis* K1 α-amylases (sequence identity with the template α-amylases from *Geobacillus stearothermophilus* is 88.33%). Calcium ion is highlighted in gray.

The α-amylase gene was 1842 bp in length, encoding a protein of 613 amino acids, including a predicted signal peptide at the amino acid position 1–28. The calculated molecular weight of α-amylase (without signal peptides) was 69 kDa, and its isoelectric point was 7.12. Functional domain annotation predicted eight stranded α/β barrels containing the active site, interrupted by ~ 70 amino acids, C-terminal domain, and calcium-binding domain protruding between β-strand 3 and α-helix 3 (Figs. 1 and S3).

**Figure 2:**
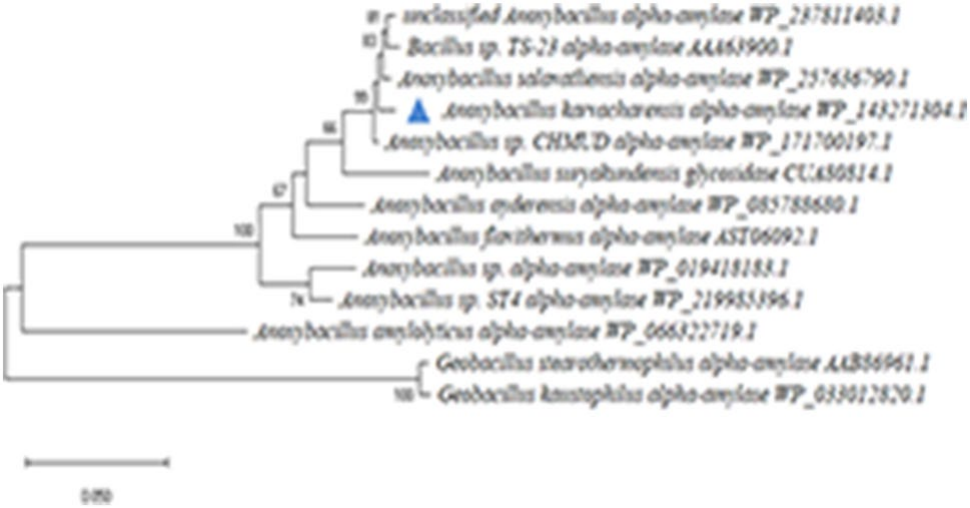
Phylogenetic relationships of α-amylases from different microorganisms. α-Amylase from. *A. karvacharensis* K1 is mentioned in triangle. The evolutionary history was inferred by using the Maximum Likelihood method and JTT matrix-based model. The tree with the highest log likelihood (− 3529.36) is shown. The percentage of trees in which the associated taxa clustered together is shown next to the branches. Initial tree(s) for the heuristic search were obtained automatically by applying Neighbor-Join and BioNJ algorithms to a matrix of pairwise distances estimated using a JTT model, and then selecting the topology with superior log likelihood value.

The sequence alignment of *A. karvacharensis* K1 amylase with several α-amylases revealed 95.11–99.02% identity to homologous sequences of α-amylases present in the genomes of *Bacillus* sp., *Anoxybacillus* sp., *A. suryakundensis, A. salavatliensis, A. ayderensis*, and *A. flavithermus* (Fig. S4). To study the phylogenetic relationships of α-amylases from *A. karvacharensis* K1 and other thermophilic bacilli, a phylogenetic tree was constructed (Fig. 2). The high similarity (99.02%) between the α-amylase of *A. karvacharensis* K1 and that of *Anoxybacillus* spp. indicated that the studied α-amylase has a high genus-specific identity.

The signal peptide of α-amylase was excluded from the PCR amplification. The α-amylase gene was successfully amplified using the designed primer sets (Figs. S5,S5’). The size of the amplified product corresponded to that of the predicted gene.

### Cloning, expression, and recombinant enzyme purification

The amplified gene product was successfully cloned into the p7XNH3 expression vector and heterologously expressed in *Escherichia coli* BL21 (DE3) cells as a soluble N-terminal His-tagged protein. After cloning and transformation, positive clones with the correct sequence of the inserted gene were determined by colony PCR and sequence analysis, and several were chosen for expression and purification of the enzyme. The α-amylases was induced using by low concentration of IPTG at 18 °C to prevent the formation of inclusion bodies.

**Figure 3:**
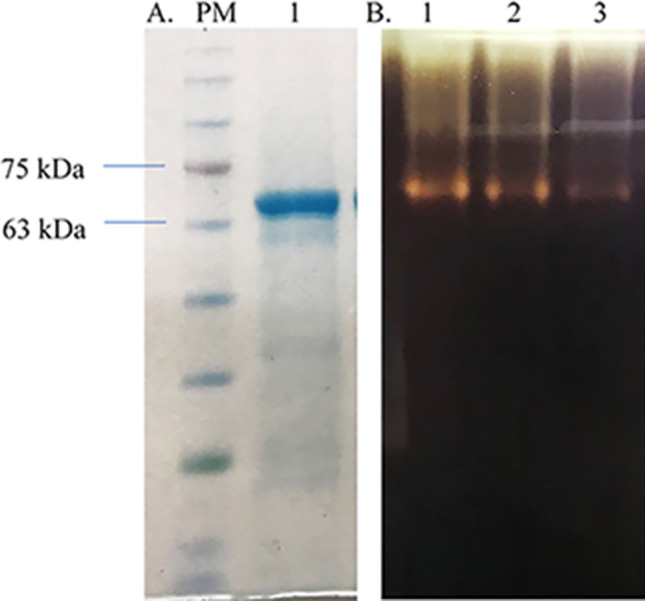
(**A**) SDS-PAGE (PM-Triple Color Protein Marker, 1—recombinant α-amylase) and (**B**) Zymogram of α-amylase in native PAGE (recombinant α-amylase loaded 1–15 μl, 2–10 μl and 3–5 μl).

Approximately 69 kDa molecular weight of the α-amylase was found by the SDS-PAGE analysis This finding was in accordance with the theoretical molecular mass calculated based on the amino acid sequence (Figs. 3, S01). High levels of recombinant gene expression were obtained in the IPTG-induced *E. coli* cell cultures. The expression differences observed in IPTG-induced cultures compared to non-induced ones were clearly indicated. Recombinant α-amylase was purified to near homogeneity in a single step using nickel affinity chromatography (Figs. S6,S6′). The obtained protein, harboring a C-terminal 6 × His affinity tag, was purified via affinity chromatography. Recombinant α-amylase was purified to homogeneity with a 1069.2 U mg^−1^ specific activity and a final yield of 38.7% (Table 1).

**Table 1:** Purification table for recombinant α-amylase.

Purification steps	Protein concentration, mg mL^−1^	Specific activity, U mg^−1^	Total protein, mg	Total activity, U	Purification fold, time	Yield, %
Crude enzyme	21.8	5.9	109.0	645.8	–	100
His-tag	0.18	1069.2	0.18	249.6	181	38.7

### Effects of temperature, pH, NaCl, CaCl_2_, metal ions, and denaturing chemicals on enzyme production

**Figure 4:**
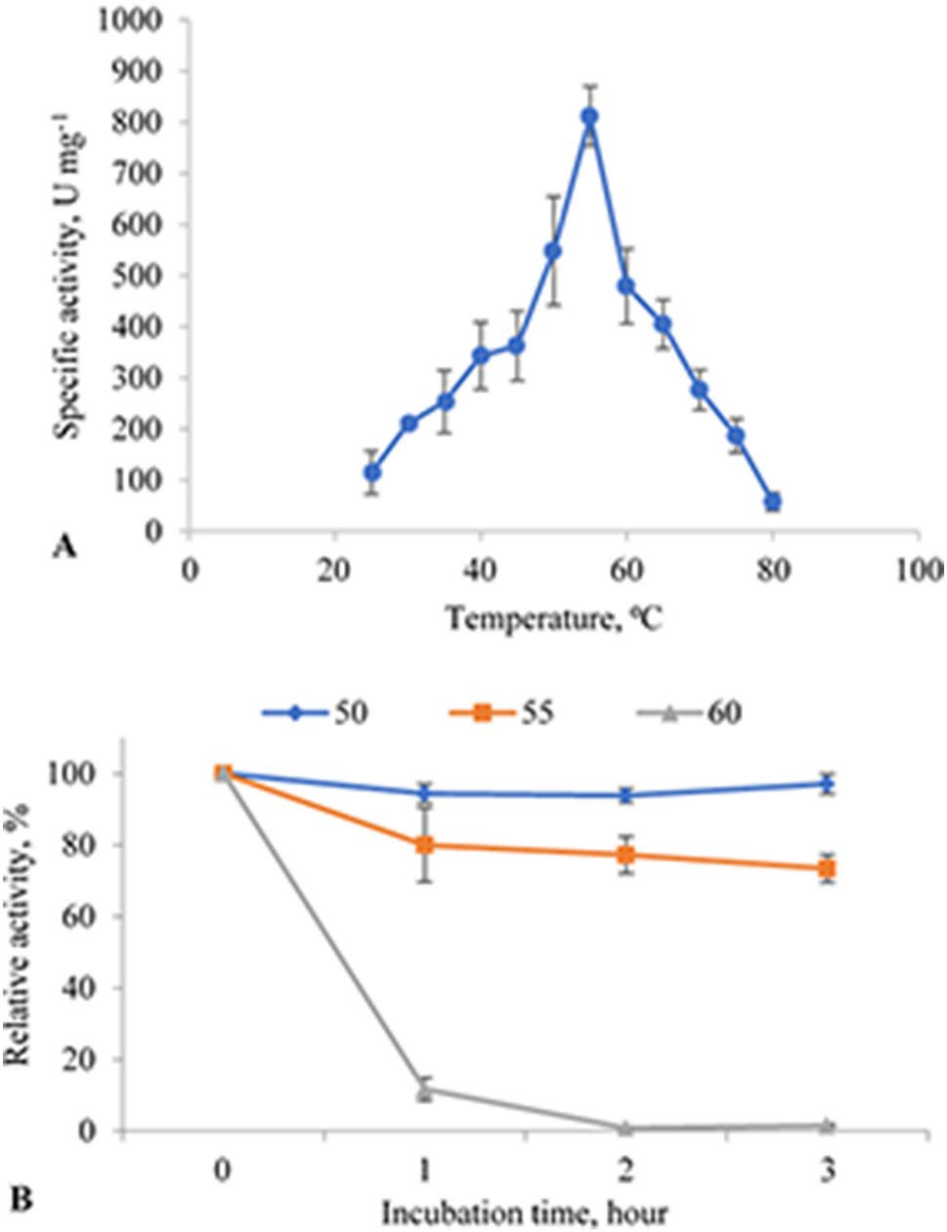
Activity and stability of recombinant α-amylase with varying temperature. (**A**) The activity of the recombinant α-amylase was assessed between 25 and 90 °C in standard assay conditions in phosphate buffer at pH 7. (**B**) Thermostability of α-amylase was studied at 50 °C (blue), 55 °C (orange), 60 °C (green) after incubation for 1–3 h. Residual activity was measured in standard assay condition in phosphate buffer (pH 6.5) at 55 °C. 100% corresponds to 1000.6 U mg^−1^.

The influence of temperature, pH, and metal ions on α-amylase catalytic activity was evaluated. The enzyme showed amylolytic activity at 25–80 °C. The enzymatic activity was increased when incubation temperature gradually increased up to 55 °C (specific activity 811 U mg^−1^) and it was decreased when temperature was further raised (Fig. 4A).

The thermal stability determination results indicated that the enzyme did not lose its activity after 3 h of incubation at 50 °C. Moreover, the enzyme exhibited more than 70% residual activity for over 3 h at 55 °C (Fig. 4B).

**Figure 5:**
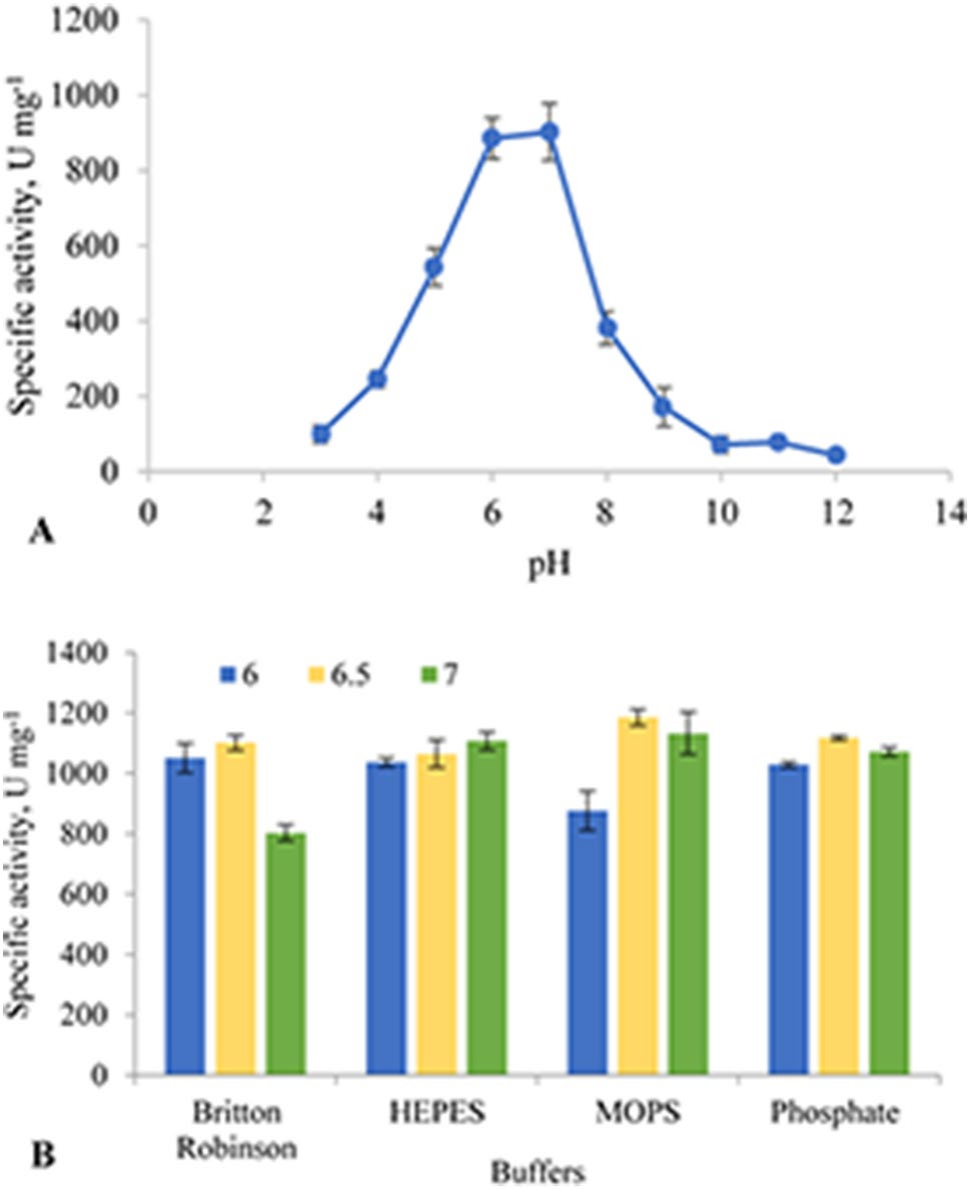
Activity of recombinant α-amylase with varying pH. (**A**) The activity and stability of the recombinant α-amylase was assessed between 3 and 12 pH at 55 °C by universal buffer. (**B**) Comparison of HEPES, MOPS and phosphate buffers with universal buffer at the pH 6.0 (blue), pH 6.5 (orange) and pH 7.0 (green). Experiments were performed with two technical replicates each from two biological replicates.

Recombinant α-amylase showed relatively high activity in the pH range of 4–9 (Fig. 5A). The enzyme exhibited maximum activity at pH 6.5. Furthermore, the effects of HEPES, MOPS, and phosphate buffers and three different pH levels (6.0, 6.5, and 7.0) (Fig. 5B) were studied. Phosphate buffer at pH 6.5 was chosen as the optimal buffer for α-amylase in further experiments. These results indicated that the enzyme was relatively active over a broad range of temperatures and pH values, making it attractive for industrial applications.

Relatively high concentrations of NaCl did not significantly inhibit the enzyme activity. Moreover, the enzyme activity increased with increasing NaCl concentrations. Enzymatic activity was observed even in the presence of NaCl (2.5 M), while the optimum concentration was 0.5 M NaCl (Fig. S7).

Ca^2+^ ions enhanced the α-amylase activity starting from 1 mM, with the maximum activity observed at 2 mM Ca^2+^ (1699.92 U mg^−1^) (Fig. S8). The metal ions did not show any significant effect on α-amylase activity (Table S1).

**Figure 6:**
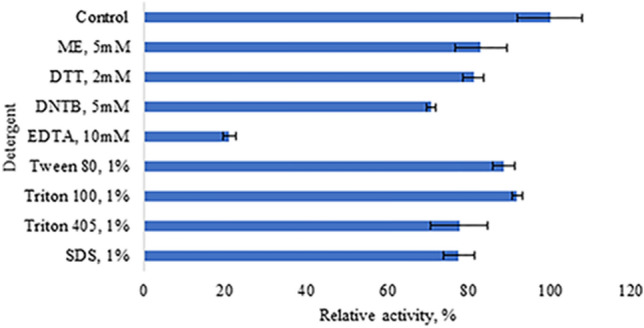
Effect of detergents and chemical compounds on the activity of recombinant amylase. The activity of the recombinant α-amylase was assessed after 1 h incubation in the presence of 1% of detergents, or 5 mM of DTNB and β-mercaptoethanol, or 2 mM DTT at 4 °C. Experiments were performed with three technical replicates. A specific activity of 1057.6 U mg^−1^ obtained without additives was defined as 100% activity.

The effects of various chemical reagents on α-amylase activity were also investigated (Fig. 6). None of the tested compounds showed noticeable inhibitory effects, with DTNB (5 mM) showing only a slight inhibitory effect. However, EDTA strongly inhibited the enzyme activity. After overnight inhibition in the presence of 10 mM of EDTA, the enzyme activity was only 20% of the initial activity. The deactivation of the enzyme in the presence of the chelating agent EDTA, clearly indicating that it is a metalloenzyme.

### Substrate specificity and kinetic parameters

The substrate specificity of α-amylase was examined against several complex carbohydrates (Table 2). A reaction mixture containing 1% of different types of starches or cyclodextrins was incubated at 55 °C and pH 6.5 for 10 min. The highest enzyme activity was observed in wheat starch at 1648.8 ± 16.8 U mg^−1^. In comparison, the highest activity towards potato starch and rice starch was 1372.6 ± 34.9 and 1278.4 ± 26.9 U mg^−1^, respectively. The specific activity of α-amylase towards amylose, amylopectin, and cyclodextrins was lower than that towards starches (Table 2).

**Table 2:** Substrate specificity of recombinant α-amylase.

Substrates	Specific activity, U mg^−1^
α-cyclodextrin	200.2 ± 8.9
ß-cyclohexane	200.2 ± 16.2
γ-cyclohexane	152.4 ± 2.1
Amylose	60.6 ± 16.8
amylopectine	1049.4 ± 5.5
rice starch	1278.4 ± 26.9
wheat starch	1648.8 ± 16.8
Corn starch	743.2 ± 28.8
potato starch	1372.6 ± 34.9
Soluble starch (control)	1069.2 ± 28.4

The α-amylase was fully maintained its activity after treatment by proteinase K.

The kinetic parameters like *K*_m_ and *V*_max_ were calculated from the Lineweaver–Burk plot using soluble starch as the substrate. Under optimal conditions (55 °C, pH 6.5), the *K*_*m*_ value for starch was 1.2 ± 0.19 mg ml^−1^, and *V*_max_ was 1580.3 ± 183.7 μmol mg^-1^ protein min^−1^.

### Analysis of the hydrolysis products

The results of HPLC analysis to determine end products of starch hydrolysis indicated that glucose and maltose were the main end products (Fig. S9).

### Utilization of whey as the substrate

AW and SW, both treated and not treated with β-glucosidase, were used to reveal the influence of different substrates on biomass and production of α-amylase. The remaining lactose concentration was calculated after the enzymatic treatment with whey. The sugar contents of two times diluted AW and SW before and after the enzymatic treatment are shown in Table S2.

**Figure 7:**
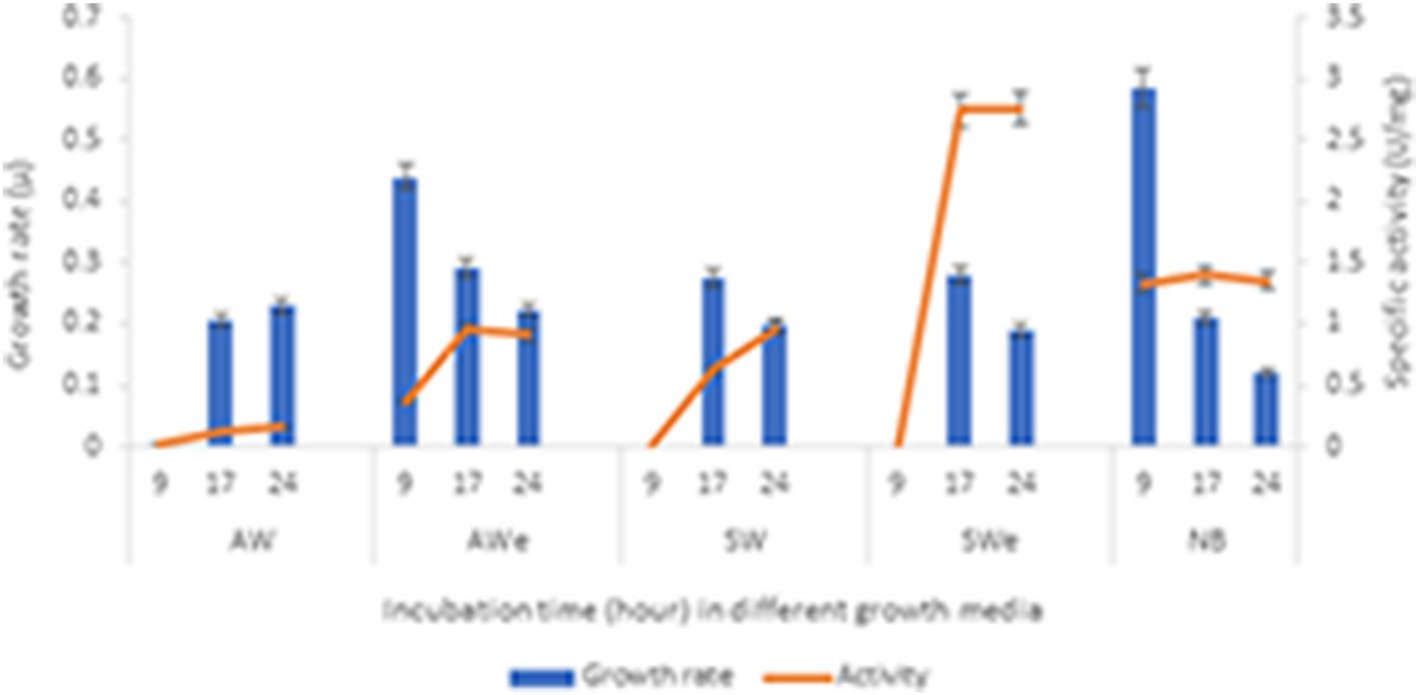
The growth rate and α-amylase activity of the recombinant amylase producing *E. coli* BL21 (DE3) on acid and sweet whey (AW and SW, correspondingly) treated and nontreated by ß-glucosidase (AWe and SWe, correspondingly) compression with NB. The growth rate and α-amylase production of *E. coli* BL21 (DE3) were followed after 9, 17 and 24 h of the incubation.

Figure 7 shows that the growth of *E. coli* BL21 (DE3) on untreated whey substrates was slow, as *E. coli* utilizes lactose at an extremely slow rate. When an enzymatic treatment was applied to hydrolyze lactose to glucose and galactose, the growth rates were much higher in both AW_E_ and SW_E_, especially in SW_E_. The α-amylase expression was higher in AW_E_ than in SW_E_. The highest yield was obtained in AW_E_ after 24 h of incubation.

## Discussion

Amylolytic enzymes are industrially important enzymes that catalyze starch hydrolysis. Many organisms can produce these enzymes, but those derived from extremophiles exhibit satisfactory properties for industrial applications. Among the thermophilic bacteria, species from the genus *Anoxybacillus* are one of the most known commercial thermostable α-amylase producers with desirable industrial features^27^.

The present study investigated an amylase-producing thermophilic bacterial strain *A. karvacharensis* K1 recently isolated from an Armenian hot spring. Although the α-amylase production by this bacterial strain quite possible to enhance by optimization of culture conditions, recombinant enzyme production offers several advantages. These advantages include easy genetic manipulation, inducible expression, fast production, high yields, and easy purification procedure. Therefore, we cloned the gene encoding α-amylase and expressed it in *E. coli* BL21 (DE3) cells, followed by purification and characterization of the recombinant enzyme. The molecular weight of the recombinant α-amylase, as determined via SDS-PAGE, was approximately 69 kDa. The purified hexahistidine-tagged α-amylase exhibited remarkable homogeneity, boasting a specific activity of 1069.2 U mg^−1^. The final yield of protein was 38.7% by nickel affinity chromatography.

α-Amylase, which has a low molecular mass, has been reported in *Anoxybacillus* spp. Thus, the molecular weights of the α-amylases of the thermophilic bacteria *A. beppuensis* TSSC-1, *A. rupiensis* TS-4, and *A. flavithennus* isolated from hot springs were determined to be 43, 48, and 60 kDa, respectively^28–30^. The molecular mass of the purified α-amylase from *Anoxybacillus* sp. YIM 342 was 68 kDa, which is equal to that of α-amylase obtained in the present study^31^.

Evolutionary analyses based on the amino acid content of the α-amylase sequence and the construction of a phylogenetic tree along with similar sequences derived from other *Anoxivbacillus* species revealed that the α-amylase from *A. karvacharensis* K1 had a high genus-specific identity.

Most of the described α-amylases require Ca^2+^ for their activity and structural integrity. These ions are important also for thermal stability^1,32^. Similarly, the 3D structural model of α-amylase obtained using the SWISS-MODEL web tool confirmed the presence of a Ca-binding domain (see Fig. 1), indicating that the α-amylase of *A. karvacharensis* K1 required Ca^2+^ for its activity. The catalytic site was located in a cleft between two domains (an [α/β]8 barrel and a large loop). The EDTA as a chelating agent had inactivating effect on enzyme, indicating that α-amylase is a metalloenzyme.

Thermozymes are optimally active and highly stable at temperatures above the host organism’s optimum growth temperature^33^. In the present study, the enzyme was active over a broad range of temperatures (25–80 °C). The recombinant α-amylase showed maximum activity at 55 °C and retained about 70% of its activity at 55 °C after 3 h incubation. It did not lose its activity after 3 h of incubation at ≤ 50 °C. The ability to catalyze reactions at moderate temperatures can broaden the application of enzymes and make them attractive for the technological and industrial sectors.

Few *Anoxybacillus sp*. that work optimally at 55–80 °C have been reported; this includes the α-amylases from *A. beppuensis* TSSC-1 (55 °C)^28^, *A. flavithermus* (70 °C)^34^, *Anoxybacillus* sp. KP1 (60 °C)^35^, *Anoxybacillus thermarum* A4 (70 °C)^36^, *Anoxybacillus* sp. AH1^37^, *Anoxybacillus* sp. YIM 342^31^, *Anoxybacillus* sp. SK3-4 (60 °C)^38^, and *Anoxybacillus ayderensis* FMB1^39^.

In the present study, the optimum pH of α-amylase was 6.5. Enzyme was stable over a wide range of pH values, from 4.0 to 9.0. At pH values below 4.0, complete enzyme deactivation was observed, probably because of the structure of the functional groups in the active site. Vieille and Zeikus^33^ reported that the acid hydrolysis of peptide bonds at low pH is a result of the cleavage of the most susceptible Asp-Pro bond. Very few thermostable α-amylases have been reported to function at high temperatures and low pH 5.0 conditions. One of them is the α-amylase of *Geobacillus* sp. IIPTN, which works at 120 °C and pH 5.0.

Another remarkable characteristic of the α-amylase of *A. karvacharensis* K1 is its tolerance against NaCl. The enzyme was active from 0 to 2.5 M NaCl concentrations. The maximum activity observed in the presence of 1.0 M NaCl. However, this α-amylase is not halophilic but a salt-tolerant enzyme, as halophilic enzymes require salt to maintain their activity and stability and deactivate in the absence of NaCl, whereas our α-amylase remained active even in case of absence of NaCl.

Metal ions and detergents did show any inhibition of the enzyme activity by more than 30%. Poli et al.^40^ also showed that the α-amylase from the thermophilic *Anoxybacillus amylolyticus* strain MR3C(T) isolated from geothermal soil (Mount Rittmann, Antarctica) exhibited high resistance to heavy metals such as Ni^2+^, Zn^2+^, Co^2+^, Hg^2+^, Mn^2+^, Cr^6+^, Cu^2+^, Fe^3+^, and Cd^2+^. CaCl_2_ at 2 mM appeared to have a slight activating effect on the α-amylase activity, increasing it 1.4 times. Industrially applicable amylases are reported to be bivalent metal ions (Ca^2+^, Mg^2+^, Mn^2+^ Zn^2+^, Fe^2+^) dependent^41^. Some metal ions like Ca^2+^ and Mg^2+^ act as co-factor which is required to increase the enzyme activity. Ca^2+^ is critical factor affecting also on thermostability^1,32^. The activation effect of Na^+^ can be elucidated by the formation of a triadic metal array, where two calcium ions flank a central sodium ion. This Ca–Na–Ca metal triad facilitates the creation of an extended substrate binding site, providing structural insights into the calcium dependency of α-amylases^42^. It was shown also inhibitory effect of some ions like Zn^2+^ and Cu^2+^ on amylase activity. . Upon Zn^2+^ binding, there is a noticeable change in the protein’s secondary structure. Modeling results suggest that Zn^2+^ forms interactions with residues H217, W244, E242, and F178 of α-amylase of *Anoxybacillus* sp. GXS-BL. These interactions prevent F178 from easily rotating and consequently inhibit the enzyme’s activity^43^. On the other hand, Cu2 + ions also induce changes in the secondary structure of the enzyme. Specifically, they increase the α-helical content and loosen the structure^44^. These ions also compete with the protein-associated cations, resulting in decreased metalloenzyme activity^40^. Resistance against metal ions makes obtained recombinant amylase prospective for industrial applications.

The structure of amylase was modeled using *Geobacillus stearothermophilus* α-amylase as a template. It has been reported that Asp232, Glu262 and Asp329 are catalytic residues^45^. It is worth mentioning that those amino acids are conserved for α-amylase of *Anoxybacillus karvacharensis* K1.

In addition to temperature, metal, and detergent stability, the enzyme was resistant to proteases, making it a potential suitable candidate not only for research, but also for applied microbiology.

Furthermore, *A. karvacharensis* K1 α-amylase showed substrate affinity towards wheat, potato, and rice starches, with hydrolysis rates of 154, 128, and 119%, respectively, and a soluble starch hydrolysis rate of 100% (Table 2). The specific activities of α-amylase towards corn starch, amylose, amylopectin, and cyclodextrins were lower than those towards the above substrates.

The production of glucose and maltose because of starch hydrolysis proved that the studied enzyme is an α-amylase.

The fields of application of recombinant enzymes are growing, paralleling the significant increase in their production volumes. Cultivation media and inducers are factors that affect the cost of recombinant enzyme production. Currently, IPTG, a lactose analog^46^, is the most widely used chemical for induction. Agro-industrial waste is an inexpensive substrate for large-scale production. Hausjell et al.^13^ showed that concentrated whey can be used instead of a defined lactose feed to produce recombinant enzyme in *E. coli* HMS174 (DE3). Similar whey milk permeates have been used for several recombinant enzymes using *E. coli* BL21 (DE3) expression hosts^47^. The utilization of lactose in whey is desirable and could be attractive for varies industrial applications. Whey contains a large amount of lactose and can be used as a culture medium to produce recombinant enzymes. *E coli*, which is widely used as an expression host, utilizes lactose very slowly. To address this challenge, enzymatic hydrolysis of whey lactose is an effective solution. By breaking down lactose into glucose and galactose, *E. coli* can efficiently utilize whey for the production of recombinant enzymes. β-Glucosidase used for that purpose is inhibited by glucose^25^. This is advantageous, as the remaining lactose can serve as an inducer to replace IPTG. After the enzymatic treatment of twice-diluted SW and AW, they contained approximately 9 g L^−1^ and 8 g L^−1^ glucose, respectively; therefore, the amounts of galactose and residual lactose were approximately 16 g L^−1^ and 25 g L^−1^, respectively. Similar to a self-inducible expression system, it is a cost-effective alternative that does not require IPTG induction^46^.

In summary, our results demonstrated that the purified thermostable recombinant α-amylase from *A. karvacharensis* K1 exhibits detergent and salt tolerance and resistance against proteases. These adaptive capabilities are important for withstanding harsh conditions usually present in industrial catalytic processes. Our results showed that whey can be used as a carbon source and inducer in *E. coli* BL21 (DE3) cultures, leading to higher growth rates and even higher α-amylase production than those using nutrient broth. The developed strategy fits the bio-based circular economy concept and is highly beneficial for the large-scale production of recombinant enzymes, as it provides a cheap substrate and a simple possibility of whey valorization. Recombinant α-amylase originating from *A. karvacharensis* K1 can be used in industrial applications, such as dishwashers and laundry detergent preparations.

## Supplementary Information

Supplementary Information.

## Data Availability

All data generated or analyzed during this study are included in this published article.
