# Supplementary material for: Detergent-resistant α-amylase derived from Anoxybacillus karvacharensis K1 and its production based on whey
Source: Sci Rep. 2024 Jun 3;14:12682. doi: 10.1038/s41598-024-63606-7 (PMC12518654; doi:10.1038/s41598-024-63606-7)
Supplement: Supplementary file 1 — Supplementary Information. [file 41598_2024_63606_MOESM1_ESM.docx]

**Supplementary materials**

**Detergent-resistant** **α-amylase derived from *Anoxybacillus karvacharensis* K1 and its production based on whey**

Diana Ghevondyan^1,2^, Tigran Soghomonyan^3^, Pargev Hovhannisyan^1,4,a^, Armine Margaryan^1,2^, Ani Paloyan^3^, Nils-Kåre Birkeland^4^, Garabed Antranikian^5^, Hovik Panosyan^1,2*^

^1^Department of Biochemistry, Microbiology and Biotechnology, Yerevan State University, Alex Manoogian 1, 0025, Yerevan, Armenia

^2^Research Institute of Biology, Biology Faculty, Yerevan State University, Alex Manoogian 1, 0025, Yerevan, Armenia

^3^Laboratory of Protein Technologies, Scientific and Production Center “Armbiotechnology” NAS RA, 0056, Yerevan, Armenia

^4^Department of Biological Sciences, University of Bergen, NO-5020, Bergen, Norway

^5^Center of Biobased Solutions (CBBS), Institute of Technical Biocatalysis, Hamburg University of Technology, D-21073 Hamburg, Germany

^a^Present address: Department of Microbiology, Biocenter, University of Wuerzburg, 97074, Wuerzburg, Germany.

***Correspondence:** E-mail: [hpanosyan@ysu.am](mailto:hpanosyan@ysu.am), Tel.: +374 94 719590; fax: +374 10 554641

**Table S1.** Effect of cations on the activity of recombinant α-amylase.

| **Metal cations** | **Relative activity, %** |
| --- | --- |
| As prepared | 100±8 |
| K^+^ | 97.4±1.8 |
| Na^+^ | 96.5±1.8 |
| Ni^+^ | 85.9 ± 6.1 |
| Co^2+^ | 92.6± 7.5 |
| Cd^2+^ | 85.7 ± 4.9 |
| Cu^2+^ | 96.0 ± 7.6 |
| Mn^2+^ | 93.7 ±3.7 |
| Zn^2+^ | 78.2 ±12.1 |
| Mg^2+^ | 94.4 ± 1.8 |
| Ba^2+^ | 111.6 ±1.9 |
| Fe^2+^ | 70.2 ±5.3 |
| Fe^3+^ | 94.2 ±4.6 |

**Table S2.** Sugar content of two-time diluted AW and SW before and after enzymatic treatment

| **Sugars** | **Before enzymatic treatment** | | **After enzymatic treatment** | |
| --- | --- | --- | --- | --- |
|  | AW | SW | AW | SW |
| Lactose (g l^-1^) | 22.1±2.7 | 26.5±2.3 | 16.5±1.1 | 25.5±1.5 |
| Glucose (g l^-1^) | - | - | 9.4±0.3 | 8.1±0.5 |

**
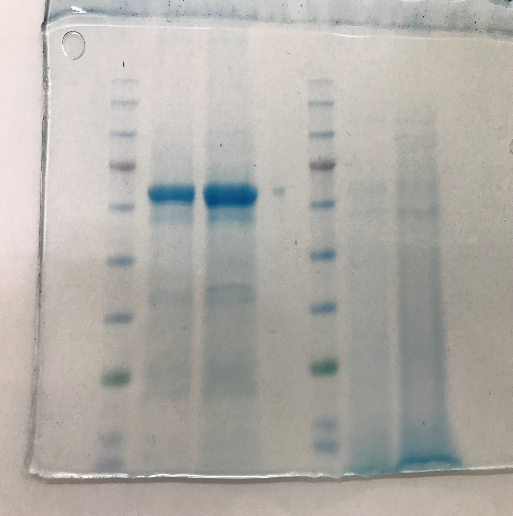
**

**Fig. S01A**. Unprocessed version of the SDS-PAGE image presented in Fig. 3 A.

**
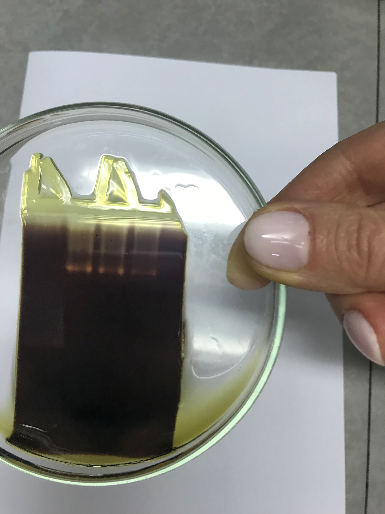
**

**Fig. S01B**. Unprocessed version of the zymogram of α-amylase in native PAGE presented in Fig. 3 B.


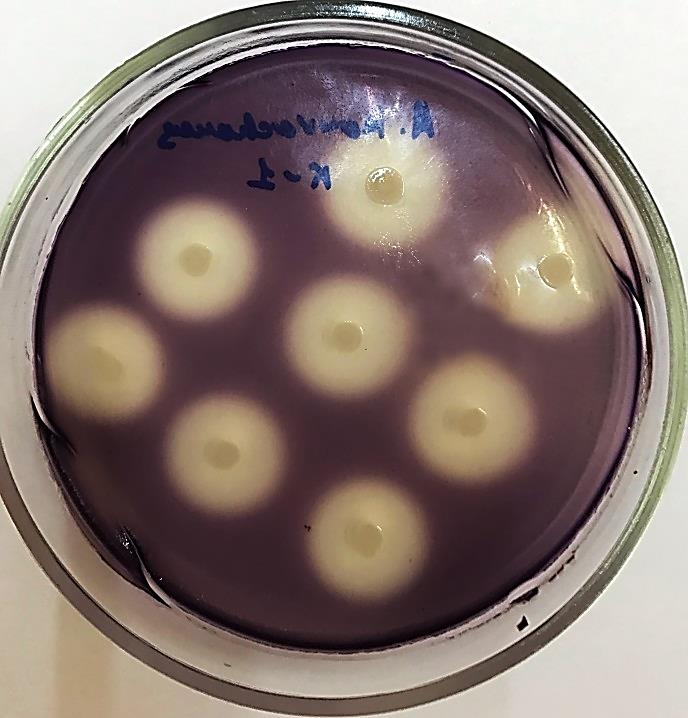


**Fig. S1.** Colonies of *A. karvacharensis* K1 showing the zone of clearance with iodine solution on starch plate.

**Fig. S2.** Time course of *A. karvacharensis* K1 growth and α-amylase production. The cells were incubated at 60°C, pH 7.0 for 18 h. The results represent the mean values of three experiments, and bars indicate standard deviation.


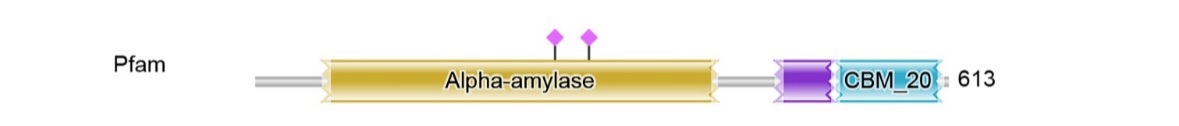


**Fig. S3.** Functional domains annotation predicted 8 stranded α/β barrel containing active site (coordinates 62-409, highlighted in yellow), interrupted by a ~70 amino acids, C-terminal domain (coordinates 462-512, highlighted in purple) and calcium-binding domain (highlighted in blue) protruding between β strand 3 and α helix 3 (coordinates 512-608).


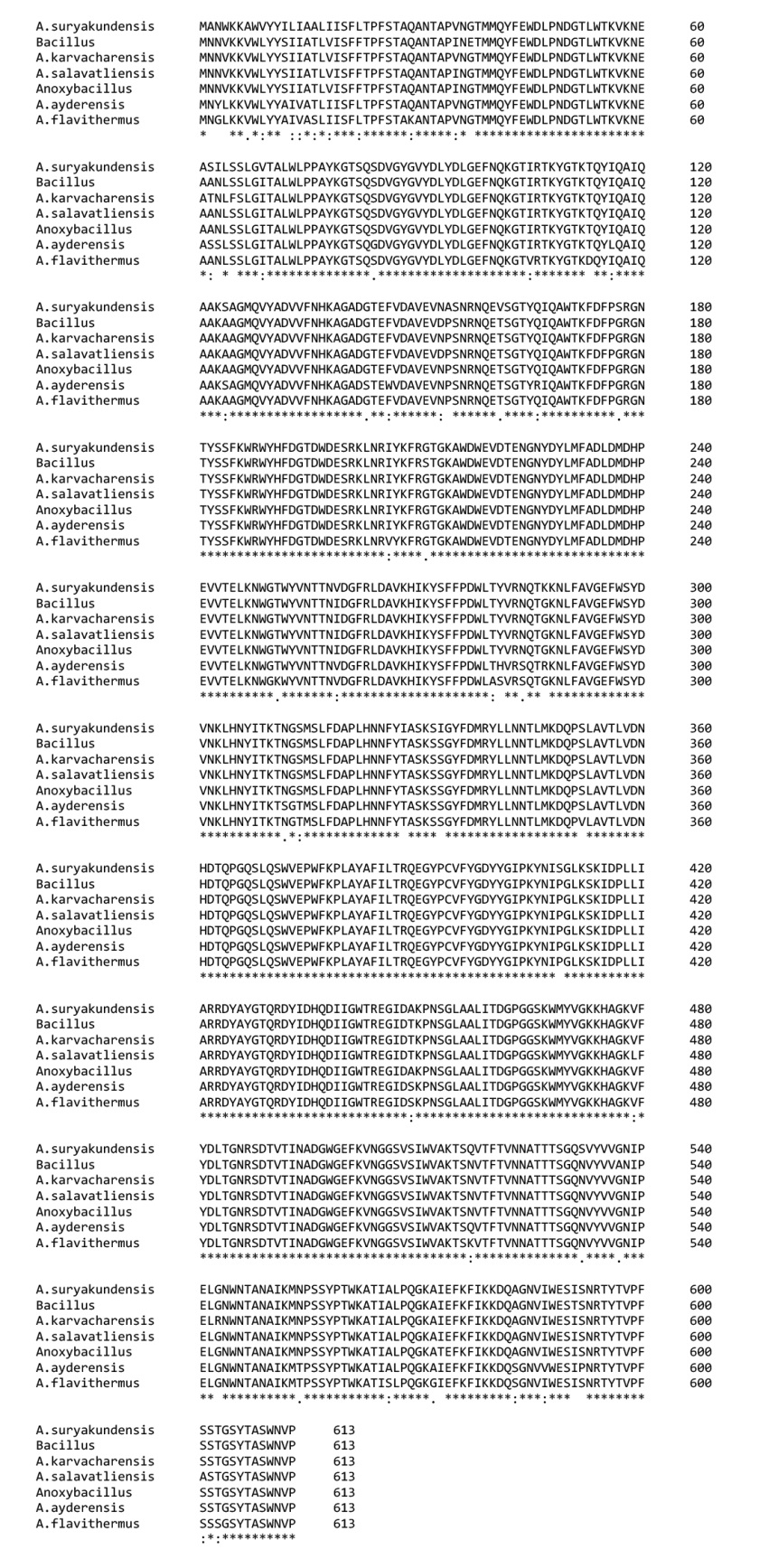


**Fig. S4.** Multiple sequence alignment of the homologous sequences of α-amylases from different sources including *Bacillus* sp., *Anoxybacillus* sp., *A. suryakundensis, A. salavatliensis, A. ayderensis* and *A. flavithermus* (*), conserved amino acids; (:), conservative replacement; (.), half conservative replacement.

**
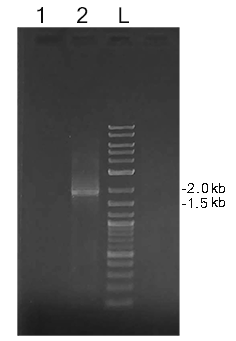
**

**Fig. S5**. The PCR amplification of the α-amylase gene (Lanes: L, 1 Kb Plus DNA Ladder; 1, α-amylase PCR negative control; 2, α-amylase gene amplification product).

**
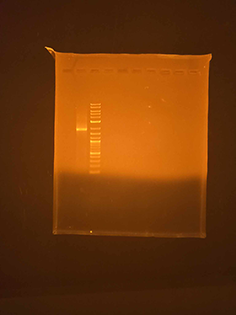
**

**Fig. S5́**. Unprocessed version of the image presented in Fig. S5.


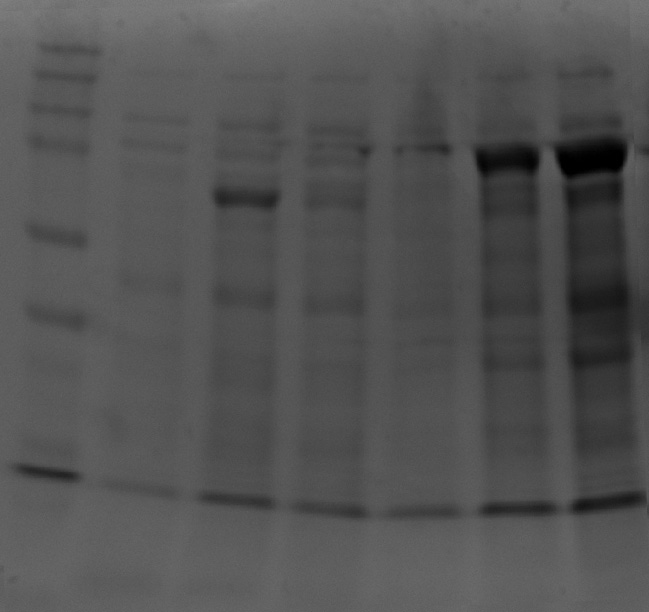


PM 1 2 3

76 kDa

52 kDa

**Fig. S6**. 12% SDS-PAGE gel of the α--amylase and purification steps (Lanes: PM, Amersham full range protein ladder; 1, non-IPTG-induced cell lysate for α-amylase clone; 2, IPTG-induced cell lysate for α-amylase clone grown 18°C; 3, IPTG-induced cell lysate for α-amylase clone grown 37°C.


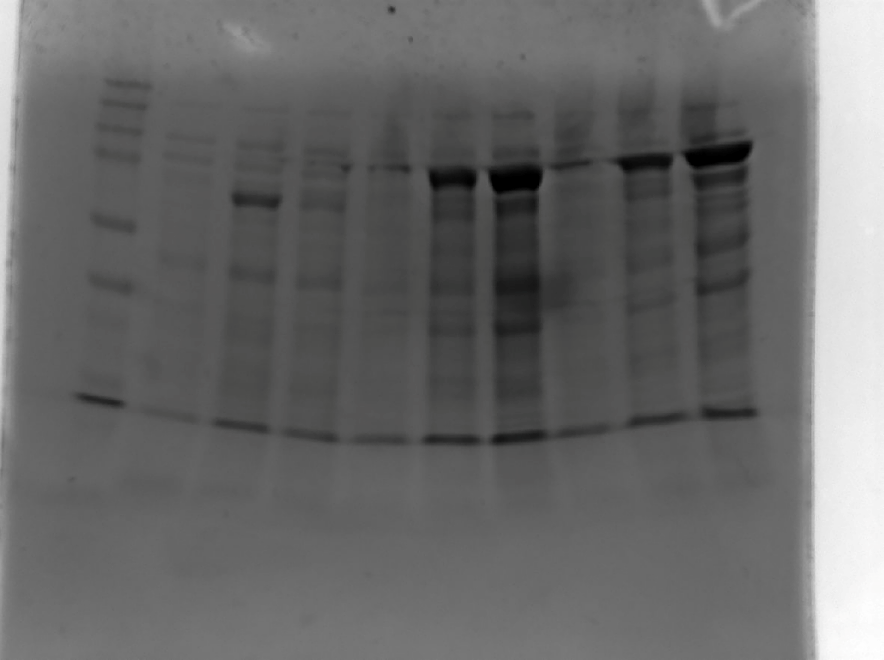


**Fig. S6́**. Unprocessed version of the image presented in Fig. S6. First four lines have been cropped to make image for Fig. S6.

**Fig. S7.** Effect of NaCl on the activity of recombinant α-amylase. The activity of the recombinant α-amylase was assessed at the presence of different concentrations of NaCl in reaction mixture. A specific activity of 1023 U mg^-1^ obtained without NaCl was defined as 100% activity.

**Fig. S8.** Effect of Ca^2+^ on the activity of recombinant α-amylase. The activity of the recombinant α-amylase was assessed with the presence of 1-10 mM CaCl_2_ in the reaction mixture.


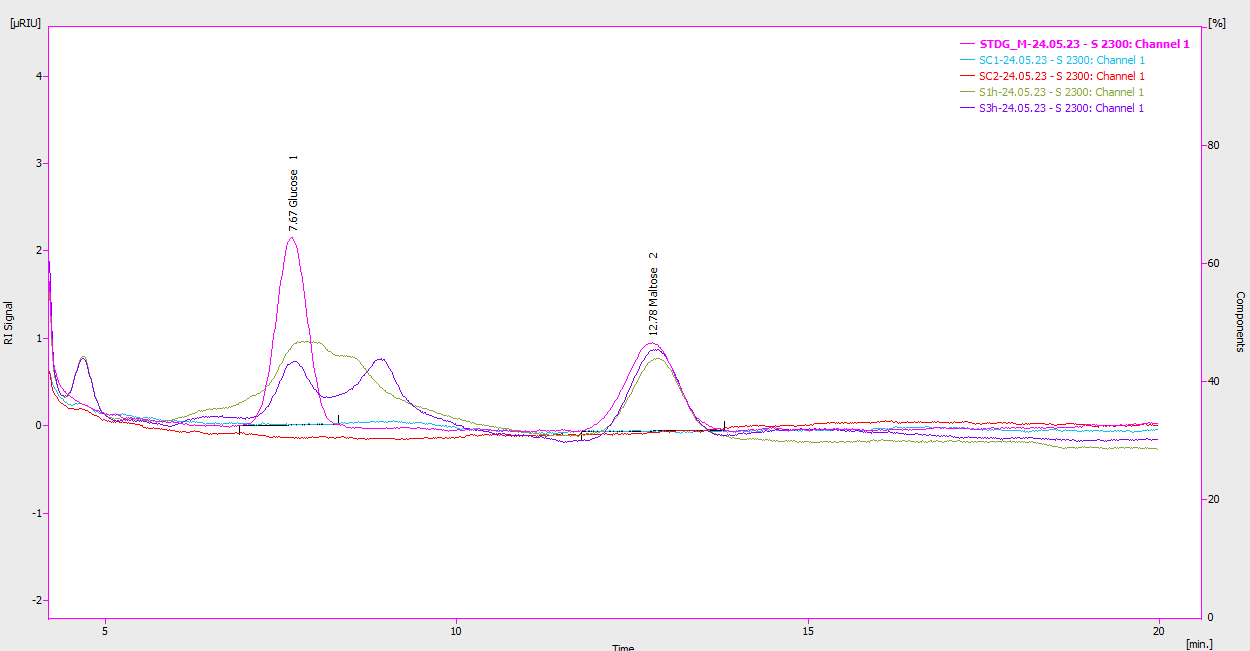


**Fig. S9.** HPLC analysis of end products of starch hydrolysis by α-amylase. STDG_M- HPLC grade sugar standard, SC1- reaction mixture without enzyme incubated for 1 h, SC2- reaction mixture without enzyme incubated for 3 h, S 1h- reaction mixture containing enzyme incubated for 1 h, S 3h- reaction mixture containing enzyme incubated for 3 h.
